# Supplementary material for: External validation of a mobile clinical decision support system for diarrhea etiology prediction in children: A multicenter study in Bangladesh and Mali
Source: eLife. 2022 Feb 9;11:e72294. doi: 10.7554/eLife.72294 (PMC8903833; doi:10.7554/eLife.72294)
Supplement: Supplementary file 1. [file elife-72294-supp1.docx]

**Supplemental File 1**

Descriptive data – demographics, predictors and viral-only outcome data from development dataset from GEMS

|  | Bangladesh  n(%)  701 | Mali  n(%)  432 |  |
| --- | --- | --- | --- |
| Age (median, IQR), months | 18 (18) | 14 (14.3) |  |
| Sex | NA | NA |  |
| Male | 407 (58.1) | 230 (53.2) |  |
| Female | 294 (41.9) | 202 (46.8) |  |
| Bloody Stool Reported | NA | NA |  |
| Yes | 500 (71.3) | 45 (10.4) |  |
| No | 201 (28.7) | 387 (89.6) |  |
| Fever Reported | NA | NA |  |
| Yes | 579 (82.6) | 299 (69.2) |  |
| No | 122 (17.4) | 132 (30.6) |  |
| Vomiting Reported | NA | NA |  |
| Yes | 215 (30.7) | 172 (39.8) |  |
| No | 486 (69.3) | 260 (60.2) |  |
| Breastfeeding | NA | NA |  |
| Yes (Partial or Exclusive) | 565 (80.6) | 283 (65.5) |  |
| No | 136 (19.4) | 149 (34.5) |  |
| MUAC (median, IQR), cm | 14.03 (1.5) | 13.77 (1.9) |  |
|  | NA | NA |  |
| Viral-Only Etiology | 145 (20.7) | 122 (28.2) |  |
| Rotavirus-only etiology | 106 (15.1) | 88 (20.4) |  |
| *Abbreviations: IQR, interquartile range; cm, centimeter; MUAC, mid-upper arm circumference* | | | |
