# Supplementary material for: External validation of a mobile clinical decision support system for diarrhea etiology prediction in children: A multicenter study in Bangladesh and Mali
Source: eLife. 2022 Feb 9;11:e72294. doi: 10.7554/eLife.72294 (PMC8903833; doi:10.7554/eLife.72294)
Supplement: Supplementary file 2. [file elife-72294-supp2.docx]

**Supplemental File 2**

List of Pathogen Targets for Taqman Array Card Testing

1. Adenovirus 40/41
2. *Aeromonas* spp.
3. Astrovirus
4. *Clostridium difficile*
5. *Campylobacteria jejuni/coli*
6. *Cryptosporidium spp.*
7. *Cyclospora spp.*
8. *Enterocytozoon bieneusi*
9. *Entamoeba histolytica*
10. *Encephalitozoon intestinalis*
11. Enteroaggregative *Escherichia coli* (EAEC)
12. Enteropathogenic *Escherichia coli* (EPEC)
13. Enterotoxigenic *Escherichia coli* (ETEC)
14. Shiga toxin-producing *Escherichia coli* (STEC)
15. *Giardia* *spp.*
16. *Helicobacter pylori*
17. *Isospora spp.*
18. Norovirus
19. *Plesiomonas spp.*
20. Rotavirus
21. *Salmonella* spp.
22. Sapovirus
23. *Shigella* spp.
24. *Vibrio cholerae*
