# Supplementary material for: External validation of a mobile clinical decision support system for diarrhea etiology prediction in children: A multicenter study in Bangladesh and Mali
Source: eLife. 2022 Feb 9;11:e72294. doi: 10.7554/eLife.72294 (PMC8903833; doi:10.7554/eLife.72294)
Supplement: Supplementary file 3. [file elife-72294-supp3.docx]

**Supplemental File 3.** Assessment of reliability and agreement between study nurses’ independent assessments of categorical predictor variables on case report forms.

|  | **Bangladesh** | | **Mali** | |
| --- | --- | --- | --- | --- |
| ***Reliability*** | κ | p | κ | p |
| Vomiting | 1.00 | <0.01 | 0.97 | <0.01 |
| Bloody Stool | 0.66 | <0.01 | 1.00 | <0.01 |
| Breastfeeding | 1.00 | <0.01 | 1.00 | <0.01 |
| ***Agreement*** | Proportion Overall Agreement (%) | | Proportion Overall Agreement (%) | |
| Vomiting | 100 | | 98.7 | |
| Bloody Stool | 98.7 | | 100 | |
| Breastfeeding | 100 | | 100 | |
