# Supplementary material for: External validation of a mobile clinical decision support system for diarrhea etiology prediction in children: A multicenter study in Bangladesh and Mali
Source: eLife. 2022 Feb 9;11:e72294. doi: 10.7554/eLife.72294 (PMC8903833; doi:10.7554/eLife.72294)
Supplement: Supplementary file 4. [file elife-72294-supp4.docx]

**Supplemental File 4.** Agreement between study nurse recording on paper case record and input into App of categorical predictor variables.

|  | **Bangladesh** | | **Mali** | |
| --- | --- | --- | --- | --- |
| ***Reliability*** | κ | p | κ | p |
| Vomiting | 1.00 | <0.01 | 0.99 | <0.01 |
| Bloody Stool | 0.80 | <0.01 | 1.00 | <0.01 |
| Breastfeeding | NA^a^ | NA^a^ | 0.99 | <0.01 |
| ***Agreement*** | Proportion Overall Agreement (%) | | Proportion Overall Agreement (%) | |
| Vomiting | 100 | | 99 | |
| Bloody Stool | 99.3 | | 100 | |
| Breastfeeding | NA^a^ | | 99.7 | |

**^a^** Data for breastfeeding did not sync successfully with the server in the Bangladesh study.
